# Gut Microbiota of Sarawak’s “Orang Ulu” Indigenous Community in East Malaysia Reveals Vanish Microbes: A Comparison With Urban Communities

**DOI:** 10.3389/bjbs.2025.15378

**Published:** 2026-01-21

**Authors:** Farhat Abjani, Yi Xian Er, Soo Ching Lee, Priya Madhavan, Anthony Rhodes, Yvonne Ai Lian Lim, Pei Pei Chong, Karuthan Chinna

**Affiliations:** 1 School of Biosciences, Faculty of Health and Medical Sciences, Taylor’s University, Subang Jaya, Selangor, Malaysia; 2 Department of Parasitology, Faculty of Medicine, Universiti Malaya, Kuala Lumpur, Malaysia; 3 School of Medicine, Faculty of Health and Medical Sciences, Taylor’s University, Subang Jaya, Selangor, Malaysia; 4 Digital Health and Medical Advancement Impact Lab, Taylor’s University, Subang Jaya, Selangor, Malaysia; 5 Institute of Biomedical Science, London, United Kingdom; 6 Faculty of Business and Management, UCSI University, Kuala Lumpur, Malaysia

**Keywords:** 16S rRNA gene sequencing, BloSSUM taxa, gut microbiota, Sarawak indigenous, VANISH taxa

## Abstract

**Introduction:**

Urbanization often correlates with reduced diversity in human gut microbiota, with notable variations observed between the gut microbiota among the Indigenous communities in rural villages and urban citizens residing in modern settings. Although research has been conducted on the gut microbiota of healthy adults in Malaysia, there has been no study characterising the gut microbiota of Sarawak’s Indigenous communities to date. This study aims to fill this gap by examining the gut microbiota profile of the Sarawak Indigenous groups (specifically Orang Ulu subethnic groups Kayan and Kenyah), comparing them with semi-urbanized Selangor Indigenous communities from Peninsular Malaysia (represented by Proto Malay subtribe Temuan) and Urban communities from Kuala Lumpur.

**Methods:**

We conducted a cross-sectional study and collected stool samples from 86 Indigenous participants from Sarawak and compared them with published data from 45 Malaysian Indigenous participants from Selangor and 18 Urban citizens living in Kuala Lumpur City. DNA was extracted from the stool samples, and subsequently, the V4 hypervariable region of the 16S rRNA gene was sequenced. The raw sequence data were analyzed using the Quantitative Insights into Microbial Ecology 2 (QIIME2) bioinformatics platform.

**Results and Discussion:**

Analysis revealed that the Sarawak Indigenous community exhibited the highest gut microbial diversity, followed by the Peninsular Indigenous and Urban groups. The *Prevotella*/*Bacteroides* (P/B) ratio revealed that the Sarawak Indigenous community showed the highest presence of *Prevotella* at 88.3%, while Kuala Lumpur Urban residents had a predominantly *Bacteroides* composition at 61%. The Selangor Indigenous community also exhibited a *Prevotella*-dominant profile at 75.5%. VANISH microbes (*Prevotella*, *Faecalibacterium*, and *Succinivibrio*) were identified as dominant genera in the Sarawak Indigenous gut microbiota, contrasting with the BIoSSUM microbe (*Bacteroidaceae*) found in the Kuala Lumpur cohort.

**Conclusion:**

This study sheds light on the distinct gut microbiota composition of Sarawak’s Indigenous community, which has not been previously explored. It highlights the impact of urbanization on gut microbiota composition during lifestyle transitions.

## Introduction

**FIGURE 1:**
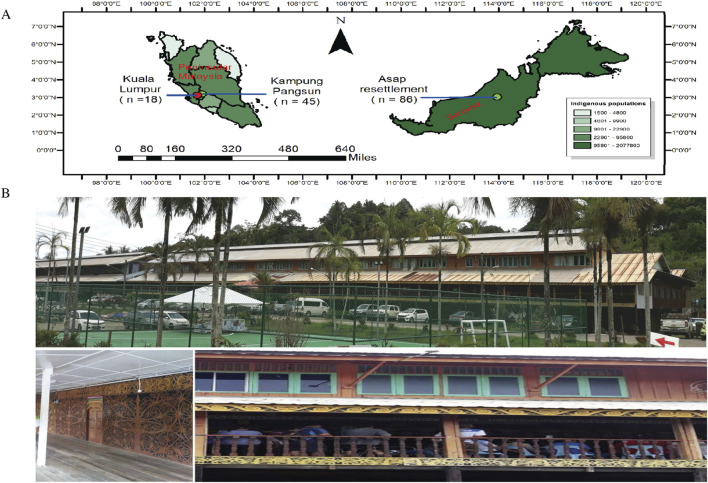
Map for East and West Malaysia and Longhouse in Sarawak at recent times. **(A)** The map illustrates East Malaysia and West Malaysia, showing the specific sampling locations. For West Malaysia, samples were collected from Kuala Lumpur and Kampung Pangsun in Selangor. In East Malaysia, indigenous stool samples were obtained from Sungai Asap resettlement village, situated in Kapit division, Belaga District, Bintulu, Sarawak. The visualization displays the geographical separation between these sampling regions across Malaysia. **(B)** The photo represents the longhouse from Sungai Asap in recent times. (Source: These pictures are original pictures and were captured during sample collection for this project in the year 2019).

Human gut microbiota is linked to many essential functions of its host, and dysbiosis is associated with the development of numerous chronic conditions [1]. The geographical location [2, 3], ethnicity [4, 5], diet [6], and lifestyle [7] have all been associated with the composition of the gut microbiota [7]. Populations living in diverse geographical and sociocultural environments are always intriguing groups to study regarding gut microbiota [8]. In such a case, Malaysia presents a unique scenario, as it is a melting pot of diverse cultural traditions and lifestyles due to its multi-ethnic population, resulting in a co-existing cross-cultural society with parallel lifestyles [7]. Moreover, Malaysia comprises East and West Malaysia (Peninsular Malaysia), with the two regions exhibiting a stark contrast to each other (Figure 1A). Geographically, East Malaysia is located on the island of Borneo, comprising 2 states (Sabah and Sarawak) and 1 federal territory (Labuan) and is predominantly covered by tropical rainforest [9]. Ecologically, it houses abundant and still largely unexplored biodiversity, which greatly influences the natural ecosystems and, in turn, shapes the lives of its people. Apart from this, East Malaysia is home to many Indigenous communities and ethnicities. The earliest known Indigenous community in Sarawak, referred to as the “Dayaks”, lead a communal lifestyle in longhouses (Figure 1B) influenced by a variety of socio-cultural and geographic factors. The Dayak community in Sarawak comprises three main ethnicities: the Iban, Bidayuh, and Orang Ulu [10]. The cultural diversity of Sarawak is highlighted by the unique coexistence of various ethnicities within the same state, similar to Sabah (with ethnicities such as Kadazan-Dusun, Chinese, Bajau, Malay, Bugis, Murut) and sets them apart from other states in Peninsular Malaysia. The Indigenous communities of Sarawak living in distinct environments are still underrepresented, and there is still no available information about their microbiota, whether in the gut, oral cavity, or nasal passages. Urbanization is associated with a reduction in microbial diversity of the human gut microbiome. Significant variations are found in the gut microbiota of people from indigenous communities and those living in modern lifestyles and environments [11]. Transitioning to an industrialized lifestyle can lead to a decline in various taxonomic groups that may be attributed to reduced consumption of microbiota-accessible carbohydrates (MACs) in the diet [11]. These taxonomic groups are known as VANISH (volatile or related negatively to modernized societies of humans), referring to the disappearance of certain microbes from the human gut, particularly in industrialized populations, and comprise microbes mainly from these families, *Prevotellaceae*, *Spirochaetaceae*, and *Succinivibrionaceae* [12]. These VANISH microbes, which encode various carbohydrate-active enzymes (CAZymes), can break down complex carbohydrates originating from plants that are high in fiber [11, 12]. In addition, urban lifestyles, which are often associated with higher frequency antibiotic exposure from food and water [13–15], decrease the abundance of VANISH microbes in the gut microbiota. People who lead modernized lifestyles are more likely to have bacteria from other families, such as *Bacteroidaceae*, *Enterobacteriaceae*, and *Verrucomicrobiaceae*, and are known as BIoSSUM (bloom or chosen in societies of modernization) [11, 12].

The impact of modern urbanization and its contribution to the rise of contemporary diseases has highlighted the urgent need to study the microbiota of unmodernized indigenous communities in the world, offering valuable insights into the human microbiome before urban influence. In Malaysia, studies that were reported on the gut microbiota mostly were focused on parasitic infections [16–19], and modern diseases include acquired immunodeficiency syndrome (AIDS) [20], inflammatory bowel disease (IBD) [21], and mucositis [22]. To our knowledge, this is the first study (to date) conducted among the Sarawak Indigenous “Orang Ulu” community to understand the interactions of human gut microbiota with their environment and lifestyle. Based on this, the main aim of this study is to compare the gut microbiota of the Sarawak Indigenous (represented by Orang Ulu: Subethnic groups: *Kayan* and *Kenyah*) with the Peninsular Malaysia’s Indigenous (represented by Proto Malay: Subtribe: Temuan) and Urban communities using 16S rRNA gene sequencing. The present study’s findings reveal the first gut microbiota dataset from the Indigenous community (Orang Ulu) of Sarawak, which enriches the current understanding of the gut microbiota of this Indigenous community. Furthermore, comparative analysis enables the identification of potential “rural beneficial microbiota” and their possible protective roles in preventing the onset of modern diseases, as well as in supporting overall human health and physiological balance.

## Materials and Methods

### Ethics Statement and Consent to Participate

The Human Ethics Applications for the study using stool samples from the Sarawak Indigenous population have been approved by the Human Ethics Committee of the National Medical Research Register (NMRR) (NMRR-17-3055-37252) and Taylor’s University Human Ethics Committee (HEC-2020/037). The methodology for this study has been designed as per the guidelines and suggestions by researchers at the University Malaya (UM) and Taylor’s University (TU), Malaysia. Moreover, before visiting the longhouse and sample collection, permission was obtained from the Tok Batin (chief of the village/longhouse).

The consent forms were given to the participants in two languages: Bahasa Melayu and English. Following the explanation, written informed consent was obtained from each participant. The participants were also assured that their personal information would be kept confidential, and they had the right to withdraw from the study without providing any reasons.

### Raw Data

The raw data of microbial 16S rRNA sequencing for Indigenous communities in Selangor and Urban communities in Kuala Lumpur City were retrieved from the European Nucleotide Archive with accession numbers PRJEB34956 (ERP117943) and PRJEB34957 (ERP117944) for downstream data analysis. They were compared with sequencing data from the Sarawak Indigenous samples, which we had collected and analysed.

For the Sarawak Indigenous community’s raw metadata, we have decided to release the data upon request after closely examining our consent forms. Participants from very small villages are included in the research study, and sharing of the metadata must be limited to protect the participants’ safety and privacy, as well as to show respect for their sensitive cultural beliefs and customs. Throughout the research process, we are committed to upholding the highest ethical standards and ensuring the welfare and rights of these vulnerable individuals are safeguarded.

### Study Cohort, Inclusion, and Exclusion Criteria for the Study

Three cohorts, namely: Sarawak Indigenous, Selangor Indigenous, and Kuala Lumpur Urban, were included in this study. With regard to the Sarawak Indigenous samples, the participants were villagers from longhouses in Sungai ASAP, Belaga town, Bintulu, Sarawak (Figure 1A). More specifically, these participants are known as Dayaks and belong to the “*Orang Ulu*” ethnicity (sub-ethnic groups: *Kayan* and *Kenyah*). A total of 108 stool samples were collected in 2019. Out of these, stool samples from 96 participants contained complete data/questionnaires and were sent to the sequencing company for 16S rRNA V4 hypervariable region sequencing. From these 96 stool samples, only 86 samples had cleared the laboratory’s Quality Control test and proceeded with the DNA sequencing and raw sequence data generation. The gut microbiota analysis for the Sarawak Indigenous community was based on these 86 samples. Of these 86 samples, 50 were from the *Kayan* sub-ethnic group, and 36 were from the *Kenyah* sub-ethnic group. For ease of understanding, the term Sarawak Indigenous community is used throughout the manuscript as a collective term for *Kayan* and *Kenyah*. Additional general characteristics of the study participants are provided in the supplementary data. For this study, the gut microbiota of the Sarawak Indigenous community (cohort) was compared with two other cohorts: Selangor Indigenous and Kuala Lumpur Urban communities from West Malaysia. The Selangor Indigenous samples (*Orang Asli*) were from the *Temuan* subtribe, a village in the Hulu Langat district in Selangor, as mentioned by Lee et al. 2019. For the Urban control, data from Kuala Lumpur City were included [19].

Participants with normal health status as indicated in their medical history records (from the local clinic/*Klinik Kesihatan*) or/and confirmed through physical examination who were also willing to comply with the study protocol, were invited to participate and provided written informed consent before being recruited into the study. As part of the exclusion criteria, individuals with a history or current presence of heavy smoking, alcohol, or drug abuse, or recent recovery from any illness within the past 3 months, were excluded from this study. Additionally, those with a history of inflammatory bowel disease or other gastrointestinal conditions within the past 3–6 months were also not eligible for inclusion.

### Collection of Dietary Patterns and Lifestyle Behaviours

An oral briefing in Bahasa Melayu (the national language of Malaysia) was provided to the participants, explaining the importance of the research and the procedures involved in the study. This briefing was conducted with the assistance of supervisors and a team of medical researchers.

At the time of stool sample collection, all the participants were interviewed to obtain their dietary patterns, lifestyle behaviours, personal hygiene, and health status. Besides that, general information regarding the participant’s height, weight, monthly income, education status, and occupation was also recorded.

### Stool Sample Collection and DNA Extraction

The OMNIgene®•GUT kit [23, 24] was used for the collection and storage of stool samples from the Sarawak Indigenous community. This kit enabled the participants to easily collect stool samples at home. Since immediate processing of the collected samples was not feasible due to the remote location, the primary purpose of using these tubes was to allow for the transport and storage of stabilized microbial DNA at ambient temperature for up to 2 months. QIAamp Fast DNA Stool Mini Kit was used to extract the DNA from the stool sample, as per the instructions given by the manufacturers [25]. The extracted DNA was stored at −80 °C until it was sent for sequencing. The V4 region of the 16S rRNA gene was then amplified using the primer pair 341F and 805R. The library was then prepared according to the Illumina 16S Metagenomic Sequencing Library preparation and amplified using the Illumina MiSeq platform with 2 × 250 bp paired-end sequencing.

### Processing of Raw Data, Gut Microbiota, and Statistical Analysis

The same analysis was conducted for all three datasets (Sarawak Indigenous, Selangor Indigenous, and Kuala Lumpur Urban). 16S rRNA sequencing reads for all three data sets for this study were processed using the QIIME2 suite of tools. The demultiplexed raw sequences (as received from the sequencing company) were processed by employing DADA2 [26], followed by removing the low-quality region and chimeras. Rarefaction plots were plotted by taking the feature frequency and Shannon diversity. A total of 149 samples were included to examine the microbial diversity. Taxonomy was assigned using the SILVA database [27]. The data generated from QIIME2 were exported as a BIOM (Biological Observation Matrix) table and subsequently analyzed using the R programming language (version 4.1.2, 2021-11-01; [28]). As the samples from all three datasets were not collected, processed, and sequenced at the same time, the 3 datasets were aggregated into a single phyloseq object and normalized via Total Sum Scaling (TSS), followed by batch correction using Batch Mean Centering [29] and ComBat modules of the Microbiome Batch Effect Correction Suite (MBECS) package. Factors, including their location, gender, and age, were tested against alpha and beta diversity indices. For alpha diversity, Shannon and Richness (observed OTUs) indices were used to examine the taxonomic diversity (richness and evenness of bacterial species) within groups. Shannon diversity index calculates the richness and evenness of various bacterial taxa [30], and richness examines the total number of species in a community (sample). Principal coordinate analysis (PCoA) was used to examine differences in bacterial communities between groups (beta diversity) employing the Manhattan, Euclidean, and Gower distance metrics. Multivariable association between 16S rRNA gene data abundances at different taxonomic levels occurring in gut microbiota (relative to location, gender, and age factors) was performed using the MaAsLin2 (Microbiome Multivariable Associations with Linear Models, with software version 2.0) [31] R package (version 1.10.0).

The alpha diversity of multiple study groups was evaluated by employing the Kruskal-Wallis statistical test, while a Wilcoxon rank sum test was employed to compare two study groups [32]. A Permutational Analysis of Variance (PERMANOVA) was used with 999 permutations to analyze the differences in beta diversity between groups of samples using the adonis function from the R package vegan version 2.5.7.0 [33, 34]. P values were adjusted for multiple comparisons tests using the Benjamini-Hochberg (BH) correction method. Basic statistical analyses and bacterial diversity plotting were performed using R Studio (version 4.1.2, 2021-11-01; [28]). The “ggplot2” package (version 3.3.5) from the R ecosystem was used for data visualization.

## Results

### Sociodemographic Features and General Characteristics of the Sarawak Indigenous Community in Sungai Asap, Bintulu

A total of 149 participants across all three cohorts were included in this study, comprising 82 females and 67 males. Participants were categorized into three age groups: 1–20 years (n = 27), 21–40 years (n = 69), and 41–60 years (n = 53). Based on the body mass index calculation (BMI), the majority of the participants (n = 46) had a normal weight. However, 29 participants were overweight, and 11 were obese/underweight.

Overall, most participants (n = 48) had lower household incomes of less than RM 800 (∼USD 172) (Supplementary Table S1). In terms of occupation, the participants were farmers, fishermen, doing some odd jobs, or students. In terms of the status of education amongst the participants, about 67.44% of them received some type of formal education, including primary, secondary, or tertiary education.

More than three-fourths of the participants (n = 70) received a government water pipe system as a source of water supply for their daily usage (Supplementary Table S1). Whereas the remaining participants had the government water pipe system along with river water (as rivers are located nearby). All the participants had a latrine facility at home. Water tanks constituted the primary type of water storage container, with direct piping systems ranking second in prevalence. Less common storage methods, classified as “others,” included plastic bottles and water buckets. About 40 participants, mentioned that they have water tanks as the main source for water storage (Supplementary Table S2). Rivers close to the settlement also serve as a source of water supply for their daily domestic requirements, from cooking to bathing and washing clothes and other daily requirements.

Dogs, cats, chickens, and pigs were the most common animals observed in the community. These animals were kept inside the cage as well as left free to move around. Based on the questionnaire and our observation, the participants have close contact with their animals. However, they normally practice using separate utensils for the animals.

Washing hands before cooking, after defecation, after contacting their animals, and after outdoor activities, boiling water before consumption, disposal of garbage at a proper place, active participation in outdoor activities, and wearing slippers before going out were found to be common and habitual practices amongst the majority of the participants.

As for the number of meals in a day, 39 participants mentioned taking three meals daily (Supplementary Table S2). Fewer participants mentioned consuming alcohol and smoking tobacco (Supplementary Table S2).

### Differences in Gut Microbial Diversity Between Sarawak and Selangor Indigenous and Kuala Lumpur Urban Communities

**FIGURE 2:**
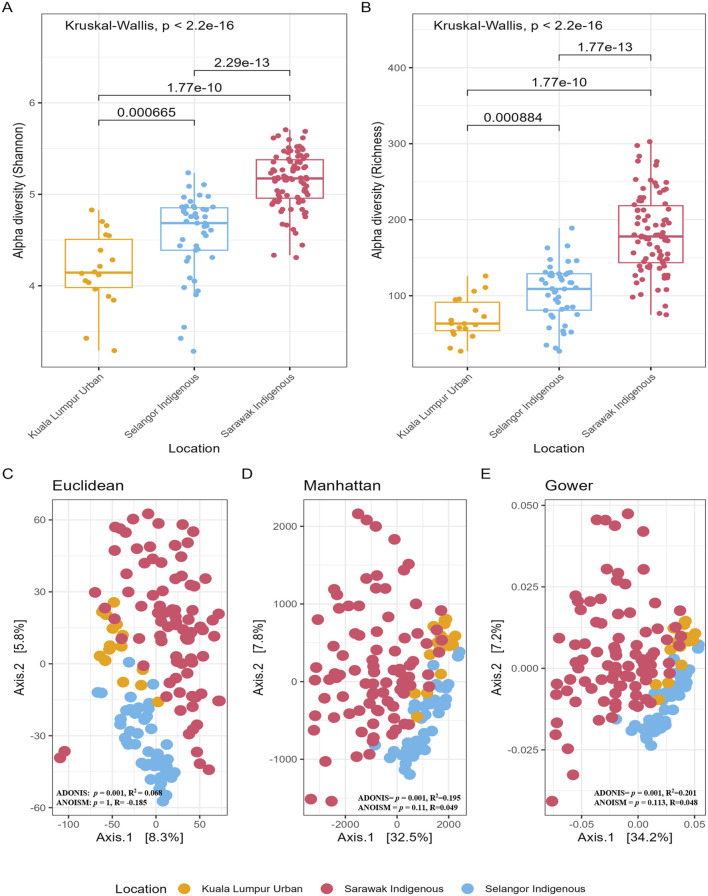
Alpha and Beta diversity of gut microbiota among Sarawak Indigenous, Selangor Indigenous, and Kuala Lumpur Urban Communities. **(A,B)** Alpha diversity boxplots of species richness and diversity based on their geographical location. A global Kruskal-Wallis test was used for statistical analysis, and a *p*-value of less than 0.05 (p-value = ≤0.05) was significant. As seen here in the box plots, the alpha diversity measuring indices show significant differences in terms of their location and age categories. **(C–E)** The figure depicts Principal Coordinates Analysis (PCoA) plots showing gut bacterial beta diversity comparisons among three Malaysian populations. Statistical analysis using Permutational multivariate analysis of variance (PERMANOVA) revealed significant differences in beta diversity based on geographic location, with a *p-value* of ≤0.05 indicating gut microbial community compositions vary significantly between these populations.

There was significantly higher species richness in the samples from the Sarawak Indigenous community compared to both the Selangor Indigenous and the Kuala Lumpur Urban communities (Figures 2A,B). Furthermore, we observed significant differences in alpha diversity indices between these communities (Shannon and Richness: Kruskal-Wallis chi-squared, *p* < 2.2e-16) (Figures 2A,B) ages in categories (Shannon: Kruskal-Wallis chi-squared, *p* = 0.033; Richness: Kruskal-Wallis chi-squared, *p* = 0.031) (Supplementary Figure S1A), but not between gender (Shannon: Wilcoxon rank, *p* = 0.46; Richness: Wilcoxon rank, *p* = 0.47) (Supplementary Figure S1B). However, age was not a significant variable for alpha diversity in the Sarawak Indigenous cohort (Shannon: Kruskal-Wallis chi-squared, *p =* 0.72; Richness: Kruskal-Wallis chi-squared, *p =* 0.72), whereas the Selangor Indigenous cohort displayed a significant difference (Shannon: Kruskal-Wallis chi-squared, *p =* 0.0032; Richness: Kruskal-Wallis chi-squared, *p =* 0.0031), this indicates that the number of microbial species in the Sarawak Indigenous cohort remains similar across all age groups (Supplementary Table S3).

There was clear clustering/segregation of the samples from Sarawak Indigenous, Selangor Indigenous, and Kuala Lumpur Urban communities (Figures 2C–E) (Manhattan distance: ADONIS, *p* = 0.001; Euclidean distance: ADONIS, *p* = 0.001; Gower distance: ADONIS, *p* = 0.001). The location of the participants played a significant role even after controlling for the age factor (Manhattan distance: ADONIS, *p =* 0.001, R^2^_Location_ = 19%; Euclidean distance: ADONIS, *p =* 0.001, R2_Location_ = 6%; Gower distance: ADONIS, *p =* 0.0031, R^2^_Location_ = 20%). No significant differences in beta diversity were observed between gender (Supplementary Figures S2A–C) and age (Supplementary Figures S2D–F), with *p-*values more than 0.05 for all the diversity indices.

### Microbial Taxonomic Comparison From Sarawak Indigenous, Selangor Indigenous, and the Kuala Lumpur Urban Communities

**FIGURE 3:**
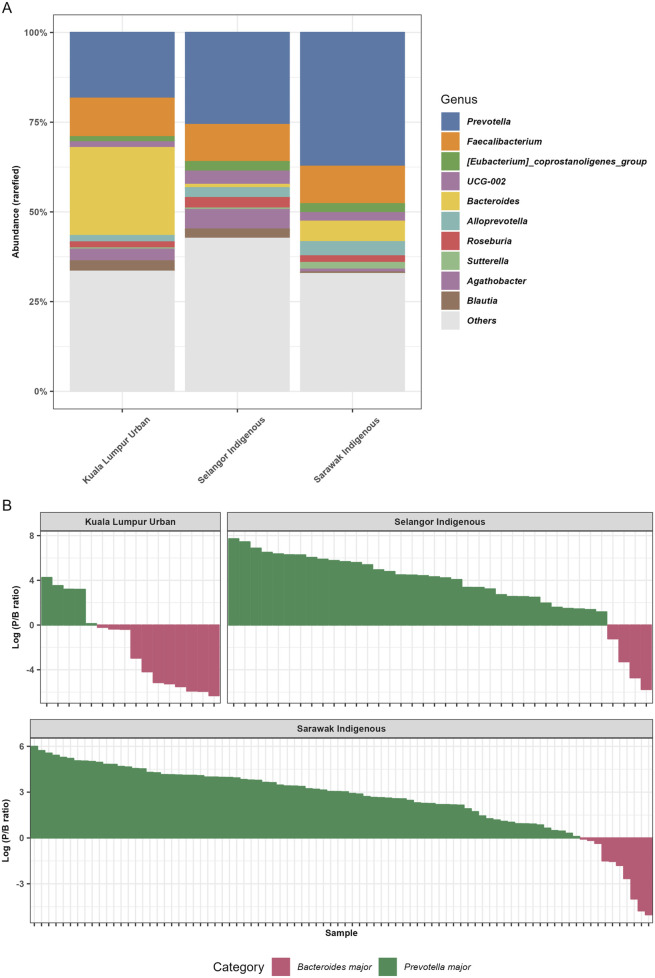
The relative abundance bar plots at the genus levels and analysis of the *Prevotella/Bacteroides* (P/B) ratios. **(A)** The figure represents the relative abundance (%) of the top 10 taxa identified at the genus level. **(B)** The figure illustrates the *Prevotella*/*Bacteroides* (P/B) ratio across three distinct Malaysian population samples. The Sarawak Indigenous community demonstrated the highest prevalence of *Prevotella* in their gut. In contrast, the Kuala Lumpur Urban community exhibited a microbiota predominantly characterized by *Bacteroides*.

The overall gut microbiota data from three Malaysian cohorts showed that *Prevotella* (41.60%), *Faecalibacterium* (16.54%), *Bacteroides* (12.10%), and *Succinivibrio* (9.83%) were the most predominant gut bacterial genera amongst Sarawak Indigenous, Selangor Indigenous, and Kuala Lumpur Urban communities (Figure 3A). Genus *Prevotella* is relatively more abundant in the Sarawak Indigenous community compared to the Selangor Indigenous and Kuala Lumpur Urban communities (Kruskal-Wallis chi-squared, *p* < 2.2e-16). However, *Bacteroides* were found to be significantly higher (Kruskal-Wallis chi-squared, *p* < 2.2e-16) amongst the Kuala Lumpur Urban community (46.8%). The relative proportions of *Succinivibrio* were more abundant among Sarawak and Selangor Indigenous communities (10.93% and 9.66%), compared to the Kuala Lumpur Urban community (1.95%) (Figure 3A). Analysis of the *Prevotella*/*Bacteroides* (P/B) ratios revealed differences among the communities studied (Figure 3B). The Sarawak Indigenous community showed the highest presence of *Prevotella* at 88.3%, while the Kuala Lumpur Urban community had predominantly *Bacteroides* at 61%. The Selangor Indigenous community also showed a Prevotella-dominant profile at 75.5% (Figure 3B).

The dominant gut microbiota among Sarawak Indigenous, Selangor Indigenous, and Kuala Lumpur Urban communities were also analysed at the Phylum, Class, Order, and Family levels (Supplementary Figures S3A–D). The microbial analysis across all participants revealed predominant Phyla including Bacteroidota, Firmicutes, and Proteobacteria, which constituted 44.78%, 40.95%, and 10.30%, respectively (Supplementary Figure S3A). Within the Class, Bacteroidia, Clostridia, and Gammaproteobacteria emerged as the principal groups, collectively representing approximately 90% of the total bacterial population (Supplementary Figure S3B). At the order level, Bacteroidales, Oscillospirales, and Lachnospirales were the dominant groups (Supplementary Figure S3C). Whereas, at the family level, *Prevotellaceae* and *Ruminococcaceae* were most abundant (comprising over half of the total families present), followed by *Lachnospiraceae* and *Oscillospiraceae* (Supplementary Figure S3D).

Finally, Supplementary Table S4 displays the PERMANOVA (Permutational Multivariate Analysis of Variance) results examining factors influencing the gut microbiome composition of the Sarawak indigenous community. The analysis uniquely identifies three statistically significant variables affecting this specific community’s microbiome: water sources (*p =* 0.045), diarrhoea (*p* = 0.014), and consumption of undercooked protein/seafood/meat (*p =* 0.048).

### The Contrasting Interactions Between the VANISH and BloSSUM Groups Between Indigenous Rural and Urban Communities

BloSSUM (observed in societies of urbanization/modernization) and VANISH (volatile and/or negatively related to modernized societies of humans) taxa are groups of microbes that are particularly linked to industrialized and non-industrialized communities, respectively [35]. We have considered the geographical locations of the participants to be indicators of the following families for this study: *Prevotellaceae*, *Succinivibrionaceae*, *Spirochaetaceae* (from the VANISH group), and *Bacteroidaceae* and *Enterobacteriaceae* (from the BloSSUM group).

**FIGURE 4:**
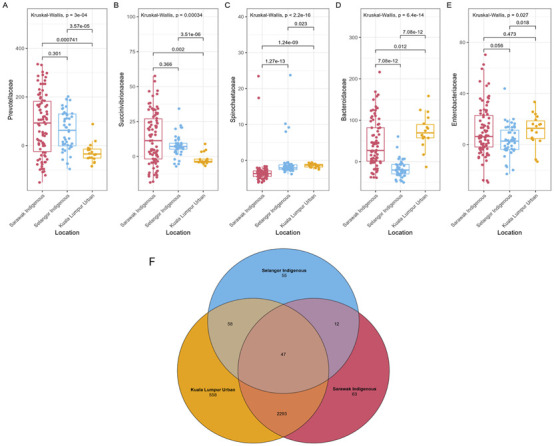
VANISH and BloSSUM gut microbiota boxplots and Venn diagram of ASVs from Sarawak Indigenous community. **(A–E)** The figure represents the VANISH and BIoSSUM taxa among the sampled populations. The VANISH taxa are composed of *Prevotellaceae*, *Succinivibrionaceae*, and *Spirochaetaceae* **(A–C)**; the BIoSSUM family taxa are *Bacteroidaceae*, and *Enterobacteriaceae* **(D,E)**. A global Kruskal-Wallis test was used for statistical analysis, and a p-value of less than 0.05 (p-value = ≤0.05) was considered to be significant. **(F)** The figure depicts unique and shared Amplicon Sequence Variants (ASVs) among different communities. 63 and 55 unique ASVs were identified for the Sarawak and Selangor indigenous communities. However, Kuala Lumpur’s urban community revealed the highest number of unique ASV, i.e. 558.

As per the analysis, the gut microbiota of the Sarawak Indigenous community showed the highest abundances of multiple VANISH microbes, which include Prevotellaceae (Figure 4A, Kruskal-Wallis chi-squared, *p* = 0.00055) and *Succinivibrionaceae* (Figure 4B, Kruskal-Wallis chi-squared, *p* = 0.00032). *Spirochaetaceae* from the VANISH family was significantly higher in Kuala Lumpur Urban than in the other two Indigenous communities (Figure 4C, Kruskal-Wallis chi-squared, *p* < 2.2e-16). Similarly, *Bacteroidaceae* from the BloSSUM family showed the highest abundance among the Kuala Lumpur Urban population when compared with the Selangor Indigenous (Figure 4D, Wilcoxon, *p* = 3.24e- 11) and the Sarawak Indigenous community (Figure 4D, Wilcoxon, *p* = 0.026). However, the difference in the *Enterobacteriaceae* family was not significantly different among Sarawak Indigenous and Kuala Lumpur Urban communities (Figure 4E, Wilcoxon, *p =* 0.457). It did exhibit a significant difference between Kuala Lumpur Urban and Selangor Indigenous (Figure 4E, Wilcoxon, *p* = 0.015) and Sarawak and Selangor Indigenous communities (Figure 4E, Wilcoxon, *p =* 0.039).

In addition, we found substantial overlap (78.1%, n = 2410) of the ASVs across the three populations sampled (Figure 4F). We found that 63 (2.60%) ASVs were specific to the Sarawak Indigenous community, 55 (31.9%) to the Selangor Indigenous community, and 558 (18.87%) to the Kuala Lumpur Urban community. Therefore, suggesting that each cohort also harbours specific microbial taxa not found in the others.

### MaAsLin2 for the Identification of Bacterial Taxa Among Sarawak Indigenous, Selangor Indigenous, and Kuala Lumpur Urban Communities

**FIGURE 5:**
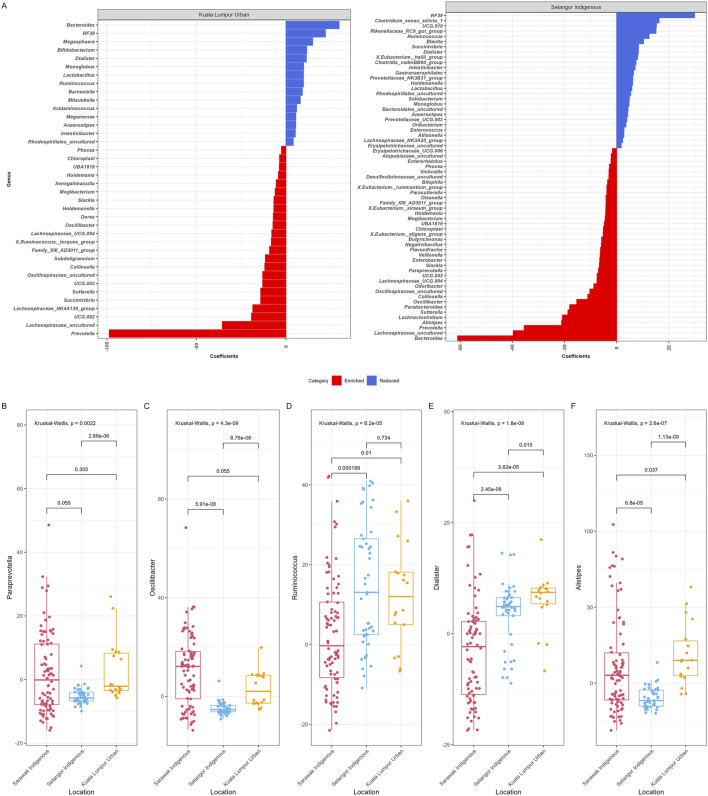
MaAsLin2 significant results. **(A)** This figure represents associations between geographical location (Sarawak and Selangor Indigenous and Kuala Lumpur Urban) and gut microbiota composition at the genus level. Based on significant results, the colour scale bar showed enriched bacteria (red) and reduced bacteria (blue) among the Kuala Lumpur and Selangor communities. **(B)** Bacterial genera based on their differential abundance among Sarawak Indigenous, Selangor Indigenous, and Kuala Lumpur Urban communities **(B–F)**.

We have also used MaAsLin2 to identify bacterial taxa (n = 75) at the genus level that were significantly associated (maximum significance = 0.05) with the geographical locations of the participants (Figure 5A), while the age group and gender were controlled. Few bacterial genera have been plotted based on their differential abundance among Sarawak Indigenous, Selangor Indigenous, and Kuala Lumpur Urban Communities (Figures 5B–F). Among all groups, the Sarawak Indigenous community had the highest abundance of *Paraprevotella*, *Oscillibacter*. The Selangor Indigenous community had the highest abundance of *Ruminococcus*. In contrast, *Dialister*, and *Alistipes* were observed in significantly higher numbers among the Kuala Lumpur Urban community (Kruskal-Wallis chi-squared, *p* < 0.05).

## Discussion

This study represents the first attempt to understand the gut microbiota profile among the Sarawak Indigenous Orang Ulu community. In this study, we presented the gut microbiota profiles of 86 Sarawak Indigenous individuals (East Malaysia) and compared them with published data from 45 Selangor Indigenous individuals and 18 Urbanites living in Kuala Lumpur City (Peninsular Malaysia). Geographically, Sarawak is home to several Indigenous communities, and almost all of them are still underrepresented. These Indigenous communities, sometimes only readily accessible by boat, provide a rare opportunity to document the influence of both the natural environment and communal living on the microbiota of its rural inhabitants that show differences in gut microbiota composition and diversity in healthy adults from Indigenous communities that are living in an environment that is close to nature and are not yet completely urbanize. Our study provided a comprehensive perspective on gut microbial analysis, revealing both expected and unexpected findings regarding differences in microbial diversity among the Indigenous communities of Sarawak and Selangor, as well as the urban community living in Kuala Lumpur. As reflected in our findings, alpha diversity was the highest in the least urbanized Sarawak Indigenous community, followed by the Selangor Indigenous community. In contrast, the urban community in Kuala Lumpur exhibited the lowest alpha diversity. These findings are in line with existing literature, where comparative analysis between Indigenous and Urban communities showed that the modern way of life and other environmental triggers may cause a reduction in microbial diversity of the human gut microbiota [36–41]. Our results are also consistent with previous studies conducted in Malaysia, which compared Indigenous and Urban communities. For example, a study conducted by Lee et al. in 2014 found that residents of the United States exhibited significantly lower bacterial diversity and evenness compared to the rural Malaysian population [18]. A recent study by Tee and colleagues shared a similar finding [17]. These findings support the hypothesis that the loss of the ancestral microbiota is linked to socioeconomic progress in society. Our research also demonstrated that urbanization is actively changing the gut microbiota of nearby populations at the expense of the disappearance and deterioration of bacteria linked to traditional lifestyles, as the least urbanized Sarawak Indigenous had the highest diversity, followed by semi-urbanized Selangor Indigenous and Urban populations. Several lifestyle changes associated with urbanization may contribute to the loss and decline of rural microbiota in urbanized populations. A complex interplay of several factors probably leads to the alterations in the microbiota of metropolitan people. Urban, industrialized populations are typically defined by modern lifestyles that involve higher consumption of processed foods [35], increased exposure to antibiotics [42], and elevated levels of air pollution [43]. These factors contribute to a decline in microbial diversity. Such reductions impair the gut microbiota’s enzymatic capacity to break down complex polysaccharides and other dietary nutrients [44], ultimately contributing to a rise in inflammatory conditions, including inflammatory bowel disease and various other non-communicable diseases that have been associated with decreased microbial diversity [11, 45]. This perhaps explains the increase in the prevalence of diabetic cases among the Malaysian population from 11.4% in 2006 to 21.2% in 2015 as urbanization and development progressed [46].

Unlike studies on general populations, the gut microbiomes of Sarawak Indigenous communities appeared to be less influenced by diet diversity and antibiotic usage. Instead, their microbial compositions were distinctly shaped by local environmental exposures and traditional dietary practices (Supplementary Table S4), highlighting how microbial influences can differ substantially between Indigenous and Urban communities. Our results also revealed that among indigenous Sarawakians, the relative abundance of gut microbiota is dominated by the members from the genera *Prevotella*, *Faecalibacterium*, *Succinivibrio*, *Bacteroides*, and *Alloprevotella* (Figure 3A).

Furthermore, our results provide additional evidence of a higher abundance of the VANISH group of bacteria in the communities with lower levels of industrialization [12, 36, 47]. Eating choices are regarded as one of the most important variables influencing gut microbiota structure because dietary fibre, fat or protein intake alters its makeup [40]. The VANISH and BloSSUM gut microbiota boxplots (Figures 4A–D) from Sarawak and Selangor Indigenous and Kuala Lumpur Urban communities reveal a higher abundance of VANISH bacteria, the family *Prevotellaceae*, in the Sarawak Indigenous cohort compared to the other cohorts (Figure 4A). On the other hand, the BLOSSUM *Bacteroidaceae* was highly abundant in the Kuala Lumpur Urban community (Figure 4D). This is also consistent with a previously reported study, where indigenous agricultural populations showed an increased abundance of *Succinivibrionaceae* and *Prevotellaceae*. Contrary, Urban populations had an increased abundance of *Bacteroidaceae* [48]. The gut microbiota of traditional Indigenous Orang Ulu may be adapted and enhanced with more anaerobic bacteria to manage the higher intake of starch, fibre, and plant polysaccharides (based on the survey). In the daily diet of Malaysians, vegetables represent a crucial food group, serving as important sources of essential vitamins and minerals [49]. Indigenous vegetables are plant species that are either native to a specific region or were introduced from other areas in the past and have since adapted and become established in the local environment [50, 51]. These vegetables can be categorized as wild, cultivated or uncultivated plants, as well as traditional and underutilized varieties. Across many rural populations worldwide, the consumption of wild plants remains a common dietary practice. In Bintulu, the intake of Indigenous leafy vegetables (ILVs) is a regular part of the local diet [51]. It is a major food supply for rural communities. The traditional practice of eating Indigenous leafy vegetables, however, is disappearing in metropolitan areas and among younger people [51]. As per our survey and the food and dietary questionnaires, a typical Kayan and Kenyah diet was rich in vegetables and vegetable leaves, including “daun ubi” (leaves of *Manihot esculenta*), “sayur manis” (Sauropus androgynus), “midin” (*Diplazium esculentum*), “daun sabong” (*Gnetum gnemon*), “daun ensabi” (leaves of *Brassica juncea* (L.) Czern. var. *Ensabi*), cucumber leaves, eggplant, and cucumber. This is evident from the 16S gene sequencing data, as our samples were rich in increased proportions of genus *Prevotella*. This genus appears to be substantially higher in abundance as a result of a vegan diet [52]. The P/B ratio revealed that the Sarawak Indigenous community showed the highest abundance of *Prevotella* at 88.3%, while the Kuala Lumpur Urban community had predominantly *Bacteroides* composition at 61%. The Selangor Indigenous community also exhibited a Prevotella-dominant profile at 75.5% (Figure 3B). The increased relative abundance of *Prevotella* in the Sarawak’s Indigenous community mainly contributed to their dietary patterns and is also similar to the other rural communities across the globe [40, 53–58]. However, as per Lee et al. 2019 [19] dietary fiber intake was significantly lower for the Orang Asli, Sarawak Indigenous population (p < 0.000), largely because the participants were mostly engaged in traditional fish rearing activities in their respective villages and, as such, had a high intake of protein from fish. Hence, their diet is quite unlike other rural and indigenous groups that have been characterized with high fiber dietary intake [19]. However, for a deeper understanding, these diet-related microbial variations between the two studied populations need to be further explored. The association between genus *Bacteroides* and dietary patterns is also associated with dietary patterns, as the *Bacteroides* were mostly associated with diets high in animal protein and saturated fat [59]. This is most likely related to their tolerance for bile, which is frequent in the gut ecosystems of people who consume animal products [59, 60]. For example, *Bacteroides* were greater in the microbiota of children in the United States who ate a Western diet versus children in Bangladesh who ate a plant-based diet [61]. Humans eating a Western diet have large proportions of *Bacteroides* in their gut, but individuals following a high-fibre diet of fruits and legumes have low proportions of *Bacteroides* [52, 55, 60–63].

Maaslin2 analysis of gut microbiota revealed distinct differences between the three cohorts studied. The Sarawak Indigenous community demonstrated notably higher abundance of several beneficial bacterial genera, including *Oscillibacter*, *Veillonella*, *Victivallis*, *Parabacteroides*, and *Paraprevotella* (Figure 5B). These microbiota appear to have significant health implications: For example, a recent study of participants from the Framingham Heart Study has shown that an increased abundance of *Oscillibacter* was significantly linked to reduced blood and stool cholesterol levels [63]. Additionally, other blood markers for reduced risk of cardiovascular disease (CVD), such as lower glucose and triglycerides and higher HDL (high-density lipoprotein), were also associated with *Oscillibacter* [63]. Intestinal *Paraprevotella* species protect the host epithelium by promoting trypsin autolysis, according to studies conducted in germ-free mice [64]. Additionally, lower intestinal trypsin levels guard against pathogen infection. Needless to say, faecal trypsin levels are typically low in healthy mice and humans [64]. *Parabacteroides* species show promising therapeutic potential in multiple conditions, including diabetes, colorectal cancer, and inflammatory bowel disease [65–69]. The distinctive dietary and lifestyle practices of the Sarawak Indigenous community may provide valuable insights for the development of microbiota-based therapeutic strategies. Our findings are consistent with the belief that communities that have experienced urbanization have a reduced variety of microbiota in their gut. Our meta-analysis based on 16S rRNA gene sequences confirmed that geographical locations, lifestyle patterns and diet are key factors that significantly shape and differentiate the composition of human gut microbial communities. We acknowledge that this study has several limitations. The lack of gut microbiota data on Sarawak Indigenous samples in the current gut microbiota database repositories may not be able to provide the most comprehensive view of their gut microbiota. Future studies focusing on recovering and characterizing these microbiota via *in silico* and/or culture-dependent methods will be needed for a more comprehensive view. Furthermore, the COVID-19 pandemic situation across the Globe was the biggest limitation, and travel restrictions imposed by the Government of Malaysia since 2020, along with the spike in COVID-19 cases throughout Malaysia, caused the unavailability of ethnicity-matched urban control samples with an equal number of participants in the same state for a better understanding and comparison of gut microbiota within Sarawak. Another limitation is that the Selangor Cohort used for comparison in this study had helminth infection, which may have altered their gut microbiota, resulting in a significant variable explaining microbial variation, and we do acknowledge that these confounding factors limit our ability to draw strong conclusions about the causes of microbiome differences between the two indigenous groups. The absence of ethnicity-matched controls and comparable urban participant numbers represents a study limitation that has broadened our research scope beyond initial parameters. Subsequent investigations involving larger Malaysian Urban populations will be necessary to further explain these preliminary findings. Moreover, it should be noted that this was pilot exploratory research that gave significant insights into the structure and execution of future gut microbiota studies in comparable settings.

## Summary Table

### What Is Known About This Subject

1. Many factors, including geography, ethnicity, diet, and lifestyle, influence the human gut microbiome.
2. Indigenous populations harbour distinct gut microbes compared to Urban populations with modern lifestyles.
3. Despite previous Malaysian gut microbiome studies, Sarawak’s Indigenous gut microbiota is still unknown.

### What This Paper Adds

1. Novel gut microbiota dataset from Sarawak’s Indigenous Orang Ulu community, previously unstudied.
2. Identification of beneficial bacteria associated with traditional indigenous lifestyles and dietary habits.
3. Communal longhouse living and natural environments help in shaping distinct gut microbial communities.

This work represents an advance in biomedical science because it reveals beneficial Indigenous gut microbiota that may help prevent the onset of modern diseases.

## Data Availability

The data that support the findings of this study are available from the corresponding author, upon reasonable request.

## Supplementary Material

SUPPLEMENTARY FIGURE S1 Alpha diversity of gut microbiota based on age and gender. Alpha diversity boxplots of species richness and diversity based on their ages **(A,B)** and gender **(C,D)**. A global Kruskal-Wallis test was used for statistical analysis, and a *p*-value of less than 0.05 (*p-value* ≤ 0.05) was significant. As seen in the box plots, the alpha diversity measuring indices show significant differences in terms of their age categories. No significant differences were observed in terms of gender.

SUPPLEMENTARY FIGURE S2 PCoA plots for gut bacterial beta diversity of ages and gender between Sarawak Indigenous, Selangor Indigenous, and Kuala Lumpur Urban communities. The Permutational multivariate analysis of variance (PERMANOVA) tests applied for beta diversity indices showed no significant differences (PERMANOVA *p* = ≤0.05) for gender **(A–C)**. However, the ages of the participants did exhibit significant differences **(D–F)**.

SUPPLEMENTARY FIGURE S3 The relative abundance bar plots and the MaAsLin2 significant bacteria. **(A–D)** These figures represent the relative abundance (%) of the top 10 taxa identified at the Phylum, Class, Order and Family levels. **(E–H)** MaAsLin2 significant Bacterial genera based on their differential abundance among Sarawak Indigenous, Selangor Indigenous, and Kuala Lumpur Urban communities.
