# Supplementary figures and images for: Gut Microbiota of Sarawak’s “Orang Ulu” Indigenous Community in East Malaysia Reveals Vanish Microbes: A Comparison With Urban Communities
Source: Br J Biomed Sci. 2026 Jan 21;82:15378. doi: 10.3389/bjbs.2025.15378 (PMC12867934; doi:10.3389/bjbs.2025.15378)

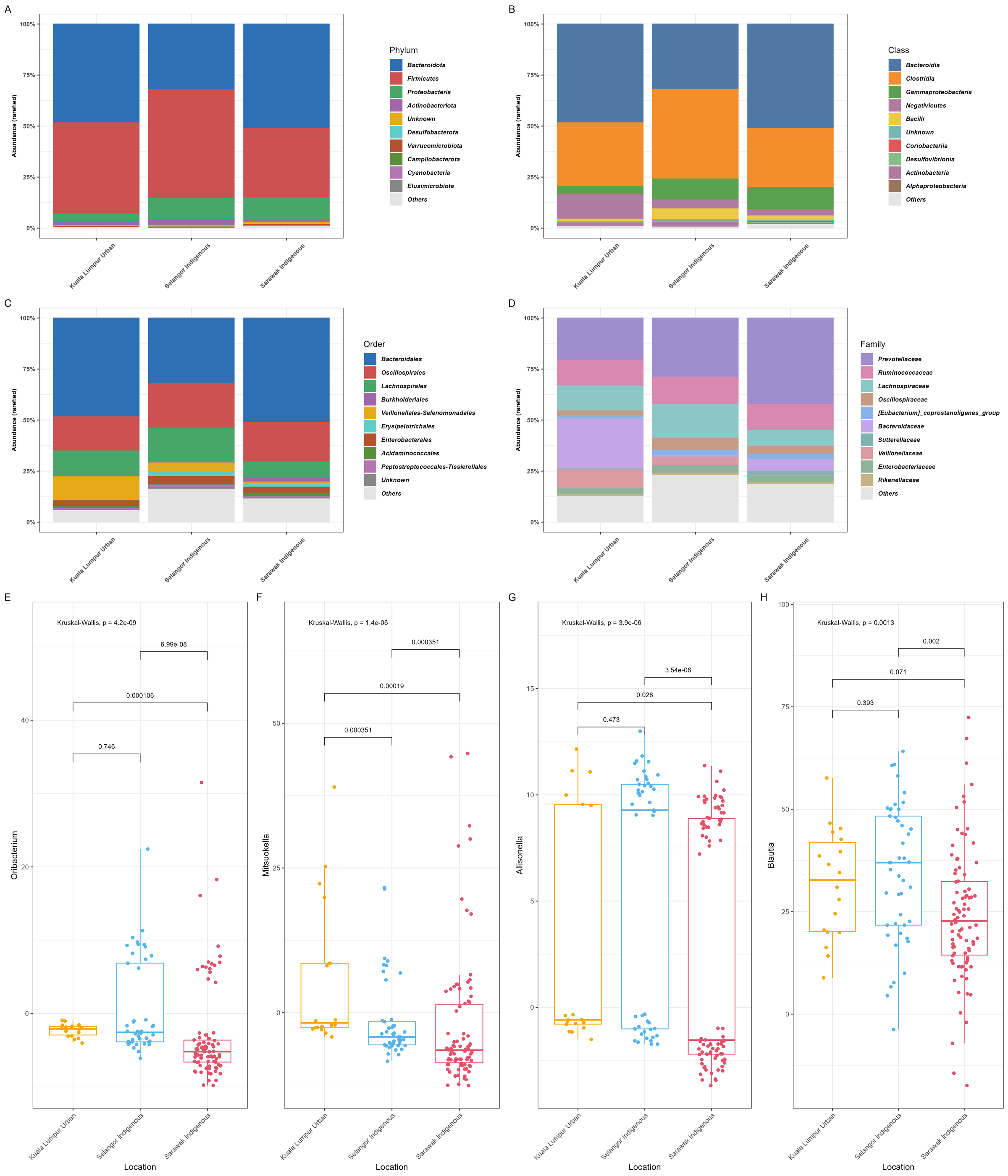

Supplement: Supplementary file 3 [file Image3.tif]

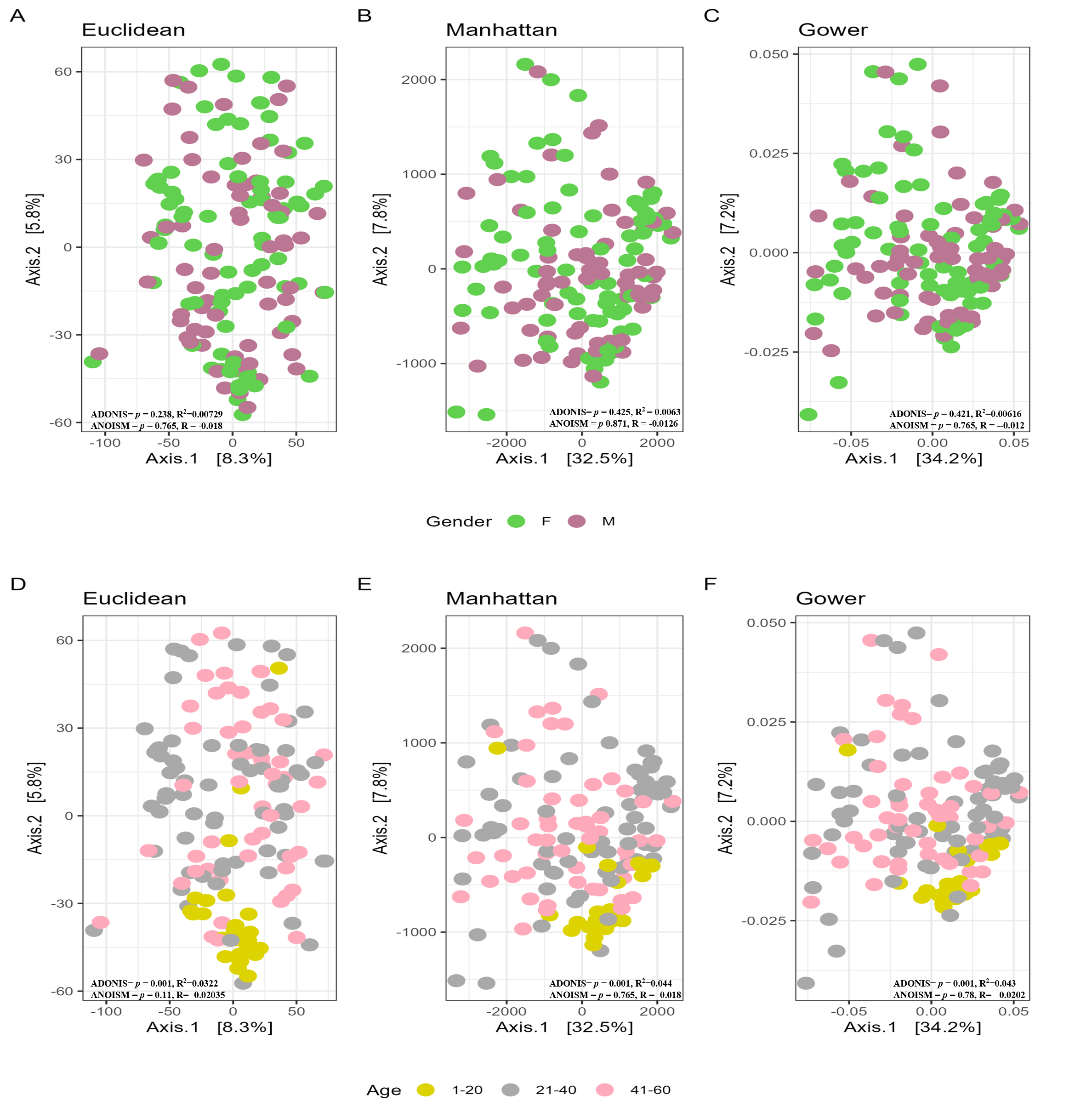

Supplement: Supplementary file 4 [file Image2.tif]

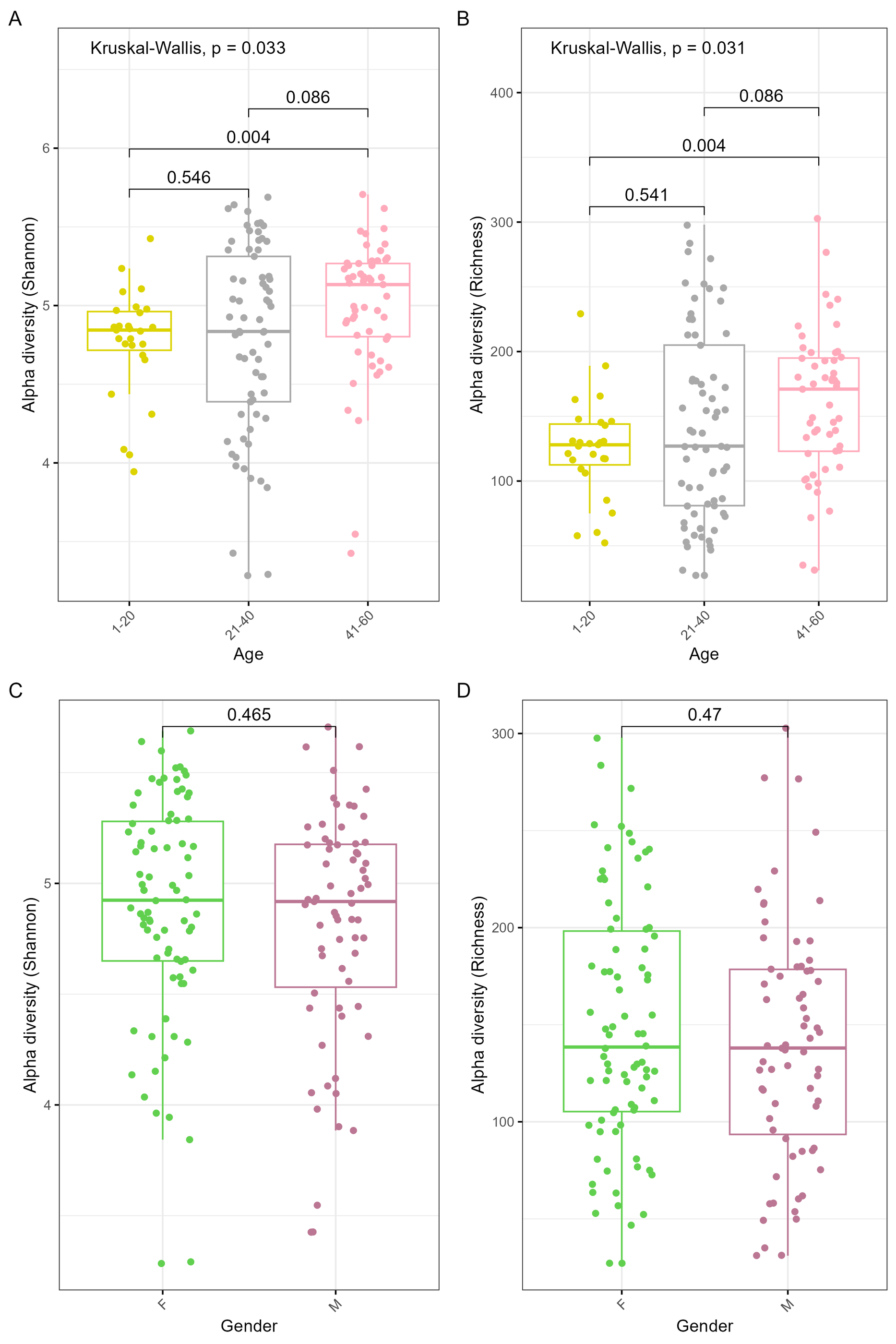

Supplement: Supplementary file 5 [file Image1.tif]
